# Perspectives on Primary Blast Injury of the Brain: Translational Insights Into Non-inertial Low-Intensity Blast Injury

**DOI:** 10.3389/fneur.2021.818169

**Published:** 2022-01-13

**Authors:** Heather R. Siedhoff, Shanyan Chen, Hailong Song, Jiankun Cui, Ibolja Cernak, David X. Cifu, Ralph G. DePalma, Zezong Gu

**Affiliations:** ^1^Department of Pathology and Anatomical Sciences, University of Missouri School of Medicine, Columbia, MO, United States; ^2^Harry S. Truman Memorial Veterans' Hospital Research Service, Columbia, MO, United States; ^3^Department of Biomedical Sciences, Mercer University School of Medicine, Macon, GA, United States; ^4^Department of Physical Medicine and Rehabilitation, Virginia Commonwealth University School of Medicine, Richmond, VA, United States; ^5^Office of Research and Development, Department of Veterans Affairs, Washington, DC, United States; ^6^Department of Surgery, Uniformed Services University of the Health Sciences, Bethesda, MD, United States

**Keywords:** low-intensity blast (LIB), mild traumatic brain injury (mTBI), behavioral abnormalities, diffuse axonal injury, synaptic alterations, mitochondrial dysfunction, neurovascular impairments, neurodegeneration

## Abstract

Most traumatic brain injuries (TBIs) during military deployment or training are clinically “mild” and frequently caused by non-impact blast exposures. Experimental models were developed to reproduce the biological consequences of high-intensity blasts causing moderate to severe brain injuries. However, the pathophysiological mechanisms of low-intensity blast (LIB)-induced neurological deficits have been understudied. This review provides perspectives on primary blast-induced mild TBI models and discusses translational aspects of LIB exposures as defined by standardized physical parameters including overpressure, impulse, and shock wave velocity. Our mouse LIB-exposure model, which reproduces deployment-related scenarios of open-field blast (OFB), caused neurobehavioral changes, including reduced exploratory activities, elevated anxiety-like levels, impaired nesting behavior, and compromised spatial reference learning and memory. These functional impairments associate with subcellular and ultrastructural neuropathological changes, such as myelinated axonal damage, synaptic alterations, and mitochondrial abnormalities occurring in the absence of gross- or cellular damage. Biochemically, we observed dysfunctional mitochondrial pathways that led to elevated oxidative stress, impaired fission-fusion dynamics, diminished mitophagy, decreased oxidative phosphorylation, and compensated cell respiration-relevant enzyme activity. LIB also induced increased levels of total tau, phosphorylated tau, and amyloid β peptide, suggesting initiation of signaling cascades leading to neurodegeneration. We also compare translational aspects of OFB findings to alternative blast injury models. By scoping relevant recent research findings, we provide recommendations for future preclinical studies to better reflect military-operational and clinical realities. Overall, better alignment of preclinical models with clinical observations and experience related to military injuries will facilitate development of more precise diagnosis, clinical evaluation, treatment, and rehabilitation.

## Introduction

The Department of Defense reported that mild traumatic brain injuries (mTBIs) comprise 82.3% of TBIs in all military branches from 2000-2021 (1), owing in large part to improvements in battlefield medicine (2, 3). Incidence rates of mTBI among military personnel are much higher than those reported for civilians (4). Low intensity blast (LIB) exposures are among the principle causes of mTBIs during military training or combat, and less frequent in industrial disasters/incidents (5–12). Service members with blast-induced mTBI have significantly higher rates of physical and mental health problems compared to soldiers with other non-neurological injuries. Awareness of potential consequences of LIB exposures has been raised by studies reporting the capacity of various types of weaponry to generate injurious blast overpressures during military and law enforcement trainings (13–15). LIB-induced mTBI injuries in Service members and law enforcement personnel have been associated with chronic subclinical effects (16). Some of these problems may not appear for months or even years after injury, resulting in lost opportunities for recognition of resulting disability and possible treatment (5, 17). Most military blast injury assessments rely on self-report in the absence of objective measurements, limiting high-quality data analysis of validated associations between primary blast exposures and long-term health effects. Preclinical models that realistically replicate the clinical pathology are needed to define the underlying mechanisms and design potential treatments (18).

**Figure 1:**
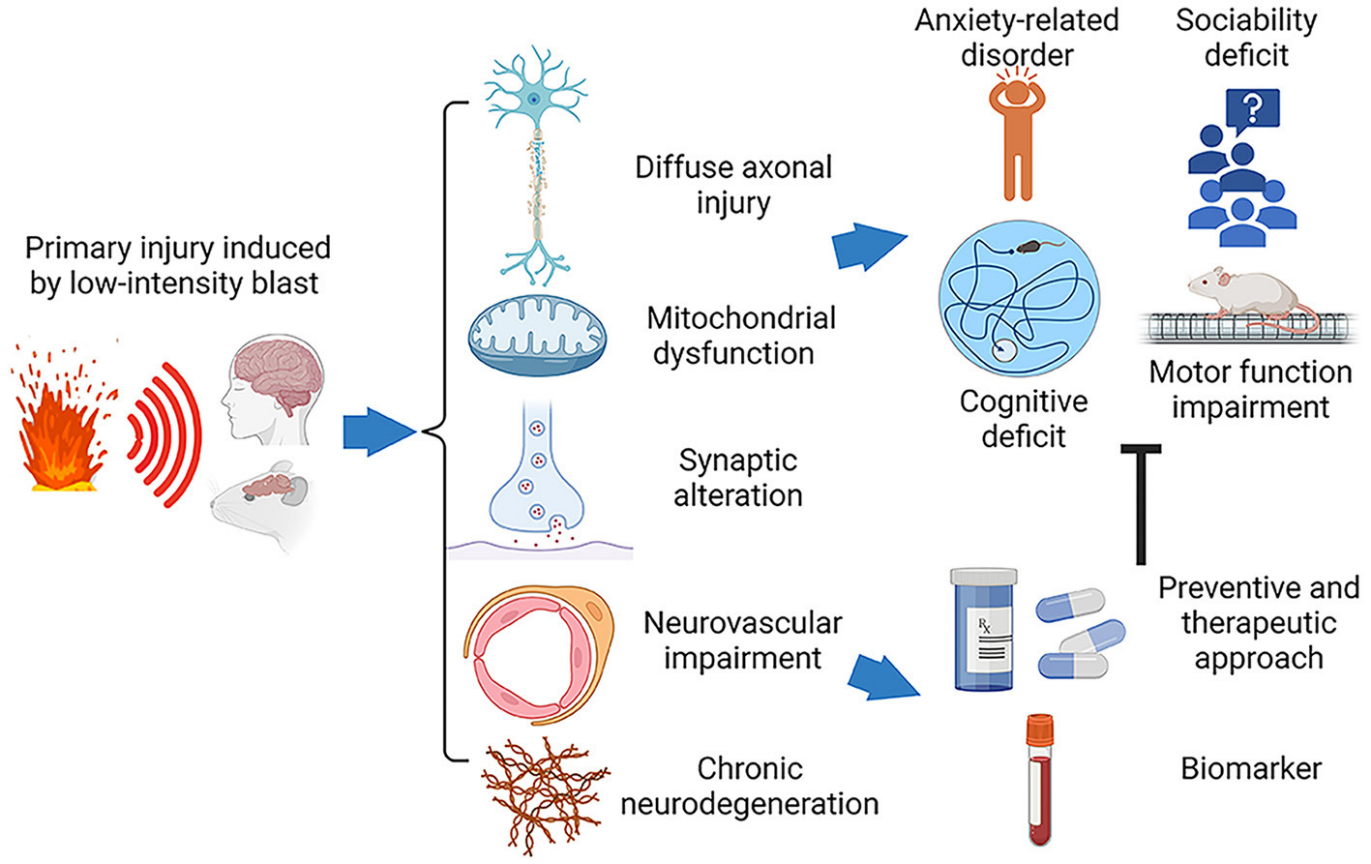
Schematic diagram of the pathophysiology and behavioral impairments in LIB-induced mTBI. Traumatic brain injury induced by primary low-intensity blast (LIB) results in cellular and subcellular deficits, including diffuse axonal injury, mitochondrial dysfunction, synaptic alteration, neurovascular impairment, and chronic neurodegeneration. These pathophysiological abnormalities lead to neurobehavioral dysfunctions, such as cognitive deficits, anxiety-related disorder, sociability deficit, and motor function impairment. The ongoing efforts targeting on these pathophysiological abnormalities to advance development of preventive and therapeutic solutions and specific biomarkers for diagnosis, prognosis and treatment.

Our prior narrative review considered blast physics, primarily based on the relationship between the intensity of blast overpressure and biological outcomes as analyzed using various preclinical models. We also introduced key components of our highly reproducible open field LIB murine model (19). We categorized non-impact, blast TBI severities into three levels based on the intensities of blast exposure: LIB impact (overpressure < 100 kPa), intermediate level blast impact (overpressure > 100 kPa to 200 kPa), and high-level blast impact (overpressure > 200 kPa). Furthermore, we demonstrated how the ground-bounce of a primary shockwave enhances the energy load by increasing impulse of the shockwave (19, 20). Our present review focuses on the effects of LIB (< 100 kPa) exposure in preclinical open-field blast studies aiming to identify research gaps and guide future research. Ideally, preclinical models should be well-aligned with clinical findings to provide valuable information on blast-induced acute and chronic pathological changes and guide the development of novel diagnostics and treatment. Figure 1 outlines methods, findings and hypotheses for preclinical studies of the non-inertial blast injury.

## Characteristics and Impact of Blast-Related mTBI in Service Members and Veterans

Extensive interagency governmental efforts support research to investigate clinical effects of military mTBI. These include the Long-Term Impact of Military-Relevant Brain Injury Consortium-Chronic Effects of Neurotrauma Consortium (LIMBIC-CENC) and the Translational Research Center for TBI and Stress Disorders (TRACTS) (21, 22). These initiatives and others confirm that Veterans and Service members with mTBI often develop psychiatric disorders, including posttraumatic stress disorder (PTSD), depression, and anxiety (22–26). Numerous clinical studies described functional impairments in participants exposed to blast(s) years before the onset of debilitating pain, increased headaches, impaired sleep, poor motor skills, and cognitive dysfunction including reduced abilities in intelligence processing speed, visual motor integration and executive functioning (22, 24, 26–28).

A study including 178,779 Veterans showed that mTBI without loss of consciousness associated with an over two-fold increased risk of dementia (29). Other studies have also implicated chronic development of neurodegenerative diseases, as well as cognitive and behavioral declines as a result of military-related mTBI (30–33). Thus, linkages between the characteristics of blast environment (intensity, number of exposure), severity of blast-induced mTBI, and chronic neurodegenerative changes continue to energize clinical and preclinical research.

Subtle transient neurologic effects following most mTBIs pose assessment difficulties in clinical practice (34). Around 50% of Veterans with blast-related mTBI report they never experienced immediate loss of consciousness, altered mental status, or temporary amnesia (22). These features of TBI are required for certain clinical assessments including the Glasgow Coma Scale, or scores on the Repeatable Battery for Neuropsychological Status (RBANS) test to classify severity of neurologic injury (35). To better understand underlying mechanisms of mild blast injury, it requires the development of “scalable, realistic, reproducible, and controllable” military-relevant preclinical models (18, 36–39). Preclinical models capable of mimicking the neurological basis of affective and cognitive disabilities in a reproduceable and reliable fashion offers the promise of improving understanding of mTBI as a spectrum disorder. This, in turn, would prompt the development of improved diagnostics and treatments. So far, after four decades of research, this promise has yet to materialize. The need exists to develop more focused and standardized scientific approaches for preclinical modeling of specific mechanisms of injury and resulting human pathology.

## Investigating Injurious Blast Forces

It is critical to delineate the physics of explosions and how physical characteristics of the generated blast relate to specific neurobehavioral and pathological outcomes of blast-induced TBI. Blast waves are formed by a sudden release of energy generated during the detonation of high-power explosives, such as trinitrotoluene (TNT) and composition 4 (C4), among others (40–42). The blast wave travels faster than sound from its center as a sphere of compressed and rapidly expanding gases. It displaces an equal volume of surrounding air at high velocity and subsequently compresses it. This overpressure phase of the blast wave is followed by a short period of negative pressure (40). As the near-spherical initial shockwave propagates, it interacts with the surrounding environment including the ground (“ground bounce”); these interactions cause reflective / refractive waves that can enhance the impulse intensity of primary shockwave (40, 41, 43).

Blast injuries are classified into four types, e.g., primary, secondary, tertiary, and quaternary (12). Primary blast injuries occur due to direct energy transfer of the shockwave (12, 18, 19, 40, 44). Preclinical blast-induced mTBI models were designed to simulate various aspects of complex blast-induced injury scenarios, including open field exposure, blast tubes using explosives, or shock tubes using compressed air or gas (38, 45, 46). Further refinement and standardization of preclinical models ideally should isolate specific aspects of primary blast components. This entails ensuring absence of head movement, burns, or impact of debris from the explosion such as occur during non-inertial or higher energy blast exposures. Open field models likely provide the most realistic blast conditions mimicking combat or military training environments. The present open-field, LIB model reproduces an operationally relevant environment including a “ground-bounce,” in addition to the standard Friedlander waveforms. The “ground-bounce” effect occurs when the explosion detonates above ground level and the shockwave hits the ground prior to impacting the subject (41, 47). This model has provided reliable and consistent findings demonstrated in previous studies (20, 41, 43, 48–50). Briefly, anesthetized mice were placed on platforms in naturally forward-facing prone position, and the LIB exposure was generated by detonating 350 grams of military-grade C4 explosive in open field. Consequently, mice positioned at a 3-m distance 1-m above ground, were exposed to 46.7 kPa (6.7 PSI) peak overpressure with a maximal impulse of 60 kPa × ms, and a primary shockwave velocity of 409 m/s (41, 43). Highly focused videography has documented the absence of head motion, which confirmed primary blast injury in the absence of tertiary blast effects (41, 43). These experimental settings replicate operationally relevant combat scenarios by providing reliable information about the key blast parameters including primary peak overpressure, maximum impulse, positive phase duration, and shockwave velocity (18, 43).

A hypothesis concerning the biologically damaging effects of the blast-body interaction posits that as blast-induced pressure waves travel through brain tissue there is excitation of the phonon continuum in brain water followed by decomposition into specific low-frequency acoustic wave oscillations (51). These oscillations surpass the tensile strength of water in the brain, resulting in rupture of subcellular structures at nanometer levels (20, 41, 51).

Our previous gross anatomical and microscopic studies of primary blast injury have shown absence of macroscopic and cell damage, including necrosis or hemorrhages in the brain after LIB exposure (19, 41). However, transmission electron microscopy (TEM) clearly delineated nanoscale neuropathology, including ultrastructural myelin sheath defects, abnormalities of myelinated axons, asymmetric synapses, neuronal soma, and dendrites (20, 41, 50). These nanoscale intracellular changes were anticipated based on the calculations of blast-induced phonon decay in brain water (51). Deployment-related open-field LIB models have reproduced other molecular alterations and behavioral abnormalities showing imputed correlations with ultrastructural findings and clinical abnormalities (52–58).

## Translational Considerations of Behavioral Impairments

### Anxiety-Related Disorders

Veterans returning from recent military conflicts may suffer from comorbidities of blast-related mTBI and PTSD, including impulsivity, anxiety, and risk-taking behaviors. These play crucial roles in premature mortality among military personnel (22–24, 26, 59). Studies conducting structured clinical interviews for the TRACTS longitudinal cohort with 450 participants from the Operation Enduring Freedom (OEF)/Operation Iraqi Freedom (OIF)/Operation New Dawn (OND) Deployments approximated a prevalence of anxiety disorder of 22.5% in blast-related military TBI, only 2.4% more than Veterans with no military TBI (22). Another multi-center clinical study from the CENC longitudinal cohort reported that a staggering 86.5% of Veterans with blast-related mTBI had been diagnosed with anxiety; this was 15% more than non-blast related individuals and 28.2% more than those with no mTBI (26). Regardless, both studies demonstrated those impacted by blast-induced neurotrauma tend to suffer from anxiety.

In our model, LIB-exposed mice (46.7 kPa) showed anxiety-like behavior in open field assessment at 3- and 6-days post injury and when tested using the light-dark box test at 5 days post-injury (41). Another study on unanesthetized rats exposed to a 95 kPa blast resulted in anxiety-like behavior assessed by elevated plus maze and acoustic startle response tests at 38- and 62 days post injury (60). However, many other preclinical studies failed to detect anxiety-like behavior, possibly due to different blast intensities or timepoints. For example, mice exposed to a 17.2 kPa blast showed no anxiety-like activity in the elevated plus maze 2 weeks after injury (54). Similar negative findings were reported in mice exposed to a 68 kPag blast by open field assessment at one- or two-weeks post-injury (61) or in mice exposed to 25 kPa blast and assessed by the elevated plus maze test as well (62). Possible reasons for differing behavioral data includes differing levels of experimenter-induced animal stress caused by handling before and during testing, duration of testing, and exposure to new testing arenas. We believe optimal evaluation of anxiety following experimental blast exposures in murine models likely resides in automated behavioral screening using home-cage monitoring approach to minimize human interference and limits animal stress.

### Sociability Deficits

Although sociability deficits occur in Service members with mTBI and in blast-exposed Veterans, preclinical studies addressing this issue are inconclusive (39, 63). Shultz et al. provides an overview of social interaction testing in the context of TBI, emphasizing the need for increased use of automated tracking capable of detecting social behaviors (64). Sociability in preclinical studies is measured by the duration of an animal's interaction with an unfamiliar animal of the same species. For example, mice exposed to ~103 kPa blast overpressure (single or repetitive) demonstrated no significant differences using a home-cage social interaction test at 3 days and 8 weeks post-injury (65). Similarly, rats exposed to higher overpressure (e.g., 150 kPa) showed no changes in social interaction-times over one-week post-injury and intact social recognition over 2 weeks post-injury (66). By contrast, mice exposed to a mild blast with only 25 kPa peak overpressure (almost five times less than in the Nonaka et al. study) displayed social interaction deficits using a two-chamber social interaction test in an open field arena at one-week post-injury (62). Interestingly, LIB-exposed mice to 68 ± 8 kPag overpressure showed stepwise development of social recognition deficits with no significant differences in social interactions during the first trial of a social recognition test at one-week post-injury. However, impaired social recognition occurred in a subsequent trial, and these deficits approached normal functioning 2 weeks post-injury (61). Further preclinical studies on the mechanisms underlying social dysfunction following LIB exposure would require delineation of methods assessing social engagement and category of responses (e.g., aggressive, or neutral behavioral responses). Such studies could use an open field arena using three-point monitoring (head, trunk, and tail) of two freely moving mice. Detailed analyses might provide better insights into treatment for post blast clinical anxiety, which seems to vary over time in affected individuals.

### Cognitive Functions

Military personnel with mTBI have impaired cognitive functioning, including poorer scores on the Weschler Adult Intelligence Scale IV coding (on processing speed), as well as worsened visual-motor integration and executive functioning (demonstrated by the Trail Making Test-B) (24). Veterans with blast-related mTBI also demonstrate slower processing speed using the National Institutes of Health (NIH) Toolbox Cognition Battery for Pattern Comparison (67). A study with 180 participants, who provided self-report measures and completed a computer-based cognitive assessment, revealed that Service members with mTBI fared worse than their control group (68). A link between self-reported PTSD and decline in executive function also occurs in Veterans with blast-related mTBI (69).

Learning and memory tests to assess cognitive impairments after blast-induced mTBI differ significantly in preclinical studies. In our initial study using a LIB model (46.7 kPa), we identified characteristic spatial learning and memory deficits using the Barnes maze (41). Numerous studies using differing levels of overpressure (39–142 kPa) showed spatial and visual recognition memory deficits from acute phase up to 2 months after blast exposures (60, 61, 70–76).

Rubovitch et al. exposing mice to 17.2 and 37.9 kPa, found spatial learning and memory deficits assessed in Y-maze 7 days after injury; these deficits persisted longer in mice exposed to higher overpressure at 37.9 kPa at 30-days post injury. Visual memory deficits measured by novel object recognition test also occurred at 7- and 30-days post-injury for both levels of blast exposure (52). A follow-up study, focusing on 17.2 kPa exposure and measured neurological outcomes 2 weeks after injury, revealed deficits of visual memory (novel object recognition test), but not spatial memory (Y-maze) or non-spatial memory (passive avoidance paradigm) (54). Since clinical observations describe cognitive impairments that develop months, years, and even decades after mTBI, preclinical research should encompass chronic time points post-injury.

### Motor Functions

Some of the Service members with mTBI demonstrate a variety of motor deficits measured by the Community Balance and Mobility Scale (77). This scale was designed based on the expertise of physical and occupational therapists to assess motor skills such as postural control during multitasking, crouching, walking sequences, dodging, running, and stopping control (78). Worsened visuomotor integration (by Trail Making Test TMT-B) has been reported in Service members and Veterans with mTBI (24). A separate study, using the Sensory Organization Test, reported chronic development of balance deficits and projected more frequent or severe deficits in Veterans with repetitive mTBI, as compared to those with single mTBI exposure (79).

Some preclinical studies using the staircase test and neurological severity score failed to find motor deficits after LIB-induced mTBI (52, 80). However, Huber et al. showed transient motor deficits in balance and gross motor coordination in mice exposed to 82-108.9 kPa overpressure, 1 h after injury; these deficits persisted until 24 h (81). Motor deficits were also observed in studies with higher blast intensities (70, 82, 83). Rats exposed to 126 kPa demonstrated motor deficits in beam-walking up to 3 days after injury (70). Mice exposed to 68 kPa displayed motor deficits assessed by rotarod test 1 and 2 weeks after injury. However, open field testing at 1 and 2 weeks after exposure did not show motor deficits (61). Mice exposed to 46 kPa overpressure traveled less distance during open field testing 3 days after injury, and in light-dark box 5 days after injury (41). Rats exposed to 39 and 110 kPa traveled less in open field at 4- and 30-days post injury (74), while rats exposed to higher overpressure (142 kPa) presented transient motor deficits in elevated plus maze. These impairments reached significance later at 44 days but not at 15- and 66-days after injury (72).

Differing approaches of generating blast conditions and differing methods for measuring neurological function and behavior could lead to contradictory preclinical findings. As already mentioned, experimenter-induced animal stress caused by handling before and during testing, duration of testing, and exposure to new testing arenas may contribute to conflicting findings. Implementing automated behavioral assessments using home-cage monitoring with minimal human interference and animal stress appears to offer more consistent results (84).

Behavioral alterations following blast-induced mTBI associate with a variety of pathophysiological changes. Our militarily-relevant LIB model showed that acute-phase affective and cognitive changes clearly associated with ultrastructural and biochemical alterations in axons, mitochondria, synapses, and ultimately neurodegeneration (19, 20, 41, 43, 48, 50). We will further consider relevant pathophysiological findings and how these may align with post blast clinical disorders.

## Pathophysiology of Blast-Induced mTBI

### Diffuse Axonal Injury

Some hypothesize that blast causes cranial deformation with compression and tension of brain tissue. These mechanical forces then generate shear forces and diffuse axonal injury (DAI) (85). In preclinical studies, axonal/myelin degeneration has been reported at overpressures > 100 kPa (83, 86, 87). In mice exposed to 46.7 kPa, silver staining revealed degenerating axon terminals in the corpus callosum, entorhinal cortex, cerebral peduncle, and fornix at 7 days, with recovery at 30 days post-injury (41). Ultrastructural axonal damage, including myelin sheath abnormalities, degenerating microtubules, and increased number of vacuoles have been found in the cortex up to 1-month post-injury (20, 41). Using systems biology analysis of gene expression, changes related to axonal and white matter degeneration were identified at 48.9 and 77.3 kPa exposures at 1-, 7-, and 30-days post-blast (88).

These findings closely align with clinical findings using diffusion tensor imaging (DTI) to identify abnormalities within white matter. DTI images showed reduced fractional anisotropy (FA) in the cerebellar peduncles, cingulate bundles, and orbitofrontal white matter in the brain of Service Members with mTBI early after injury (89). Later observations showed reduced FA in the cerebellar peduncles 2–4 years following injury (90).

### Mitochondrial Dysfunction

Mitochondria were found to be among the most vulnerable cellular organelles affected by blast exposure, although most studies used conditions of high blast intensities (91). It has been suggested that proteins involved in programmed cell death or apoptosis may cause mitochondrial swelling and exacerbate cellular injury by the cascade of pro-apoptotic pathways, such as release of cytochrome c, Bcl-2, and caspases (49, 92, 93). Mitophagy is responsible for the selective degradation of mitochondria (94). Dysregulation of mitophagy has been accompanied with TBI pathophysiology (95, 96). Mitophagy is also well implicated in Alzheimer's disease, Parkinson's disease, cerebral ischemia, multiple sclerosis, diabetes, and obesity with involvements of Dynamin-1-like protein (Drp1) (97–99). In our studies using proteome analysis of brain tissue, we reported that overpressure of 46.7 kPa caused dysregulation of fission-fusion processes underlying mitophagy, evidenced by decreased Drp1, and changes in fission protein (Fis1), inner membrane fusion protein (OPA1) and outer membrane fusion protein (mitofusin 2) at 7- and 30-days post-blast (50). Confirmatory findings included changes in mitochondria-related proteins involved in oxidative stress and mitochondrial volume-regulation. These molecular changes occurred in parallel with mitochondrial ultrastructure abnormalities, including swollen and degenerating mitochondria, at 7- and 30-days post-blast (41).

As mitochondria play a vital role in energy metabolism, these findings have translational correlates. [18F]-fluorodeoxyglucose (FDG)-positron emission tomography in Veterans with blast-induced TBI showed significantly reduced metabolic rate of glucose metabolism in the amygdala, hippocampus, and thalamus (100). These findings suggest mitochondrial dysfunction and impaired bioenergetics may underly neurological impairments (101), potentially serving as a therapeutic target for early intervention and prevention.

### Synaptic Alterations

Glutamate-dependent excitotoxicity and subsequent neurotoxicity are among most important mechanisms causing impaired homeostasis after blast exposures. However, studies on glutamatergic synapses after LIB provide conflicting data. Using TEM, we observed that LIB increased the number of excitatory synapses in the hippocampus. By contrast, structural synaptic changes were decreased in the cortex of LIB-exposed mice (46.7 kPa) at 7- and 30-days after blast (20). Deficits in hippocampal short-term plasticity and synaptic excitability have been induced by 103 kPa blast exposure at 21 days post-blast (102). In comparison, repetitive exposures of 100 kPa overpressure caused increased synaptic excitability in mice at 1-day post-injury (103). A study exposing rats to repetitive blasts at 74.5 kPa showed behavioral deficits, which were successfully mitigated by a metabotropic glutamate receptor antagonist (104).

*In vitro* studies assessing synaptic dysfunction in hippocampal slice cultures exposed to 39 and 92.7 kPa blasts showed reduced long-term potentiation (LTP), which might underlie impaired learning and memory (105, 106). This *in vitro* finding was supported by an *in vivo* mouse study showing reduced LTP 2 weeks to 1 month after a single exposure to a 77 kPa blast (107). In contrast, another study using a 103 kPa exposure showed no impairments in LTP measured at 21 days after blast (102). These contradictory findings may be attributed to differing experimental methods, various levels of blast exposures, experimental conditions, and time points of outcome measurements. Future studies aim to define dynamic changes in glutamatergic synapses to develop interventions to mitigate such pathologic changes. Ideally, such experiments would employ a multiple time-point electrophysiological measurement at acute and chronic time points using a standard open field blast model.

### Neurovascular Impairments

Decreased cerebral blood flow with associated poor white matter integrity has been described in Veterans with mild-to-moderate TBI several months after blast injury (108). Others have reported altered vascular remodeling. For instance, Veterans with chronic blast-related mTBI demonstrated altered microRNA involved in vascular remodeling (VEGF signaling) (109). Another study found significantly increased plasma levels of vascular endothelial growth factor-A in Veterans with blast-related mTBI that correlated with reduced glucose metabolism and poor cognitive inhibition (110). Overall, cerebrovascular dysfunction leading to cerebral blood flow reduction following blast-related mTBI appears capable of degrading neurovascular integrity, causing detrimental effects on neurons, and resulting neurodegeneration (111, 112).

Numerous preclinical studies have investigated the structure and functions of the neurovascular unit (NVU), which is comprised of a network of endothelial cells, extracellular matrix, pericytes, astrocytes, and neurons (113). Several studies characterized the blast-induced pathology of NVU by swollen astrocytic end feet, disrupted pericytes and endothelial cells, and abnormally shaped lumen or capillaries. For example, Sosa and colleagues (107, 114, 115) described significant vascular changes in rats exposed to repetitive LIB (75 kPa) 1 day and 6–10 months after injury. They showed that 17% of repetitive LIB-exposed rats had blood in the lateral ventricles several months later. They suggested that the presence of blood in the ventricles of LIB-exposed rats, even 10 months post-exposure was caused by chronic effects of blast on the choroid plexus vasculature. The resulting vascular fragility and epithelial damage leads to persistent blood leakage. Acutely increased permeability of the blood-brain barrier (BBB) induced by the dysregulation of NVU was reported in mice after single or repetitive exposures to 105 kPa (116). Similar findings were reported by Kawoos et al. (117) using rats exposed to single and multiple blast exposures at 72 or 110 kPa. The authors reported a significant increase in BBB permeability in various parts of the brain and a marked increase in intracerebral pressure (ICP) in all groups except the single 72 kPa blast exposure group. They suggested that the extent of ICP increase and BBB permeability change depended on intensity and frequency of blast. These observations suggest a clinical advantage in measuring and lowering ICP pressure immediately after blast exposures.

Using an open-field experimental setting to induce low-level pressure effects (17.2 kPa) to the mouse brain, Rubovitch et al. detected BBB leakage using T1-weighted MRI 30 days after exposure (52). Several authors offered suggestions concerning molecular mechanisms underlying BBB impairment after LIB exposure(s) (114). These included increased oxidative stress, MMP activation, inflammatory reaction, and increased glia cell activity. Combined qualitative and quantitative ultrastructural analyses may provide better understanding on the altered homeostasis of NVU following LIB exposure.

### Impact of Blast Injury and Related Neurodegeneration

Blunt impact mTBI increases the risk of chronic development of tau-dependent neuropathology, a hallmark of neurodegenerative diseases including Alzheimer's disease (AD) or chronic traumatic encephalopathy (CTE) (118). Whether blast-induced mTBI causes AD or CTE remains to be determined. A small cohort study including military training personnel revealed lower tau protein expression in the blood of 29 participants exposed to over 5 psi (34.47 kPa) at 24 h after exposure, and higher expression at 72 h post-blast as compared to those exposed to < 2 PSI (13.78 kPa) (119). A CENC report comparing six longitudinal studies with nearly 1,500 participants, including both active duty Service members and Veterans exposed to sub-concussive and concussive blasts, showed a range of neuropathological changes related to functional connectivity, cortical thickness, and levels of peripheral blood tau biomarkers (120). These studies found that increased incidence of military-relevant mTBI associates with increased neurodegenerative biomarkers, including exosomal tau/phosphorylated-tau that relate to cognitive, affective, and somatic post-concussive symptoms (121). Moreover, investigations into tau mechanisms have demonstrated prion-like properties of tau, where it can spread to other brain regions (122). This has been demonstrated in TBI, where regions unaffected by the insult show evidence of tau spreading (123). Amyloid β peptide (Aβ) is also associated with progressions of CTE in military Veterans with TBI (124). Increased Aβ40 and Aβ42 serum levels were reported in military and law enforcement personnel exposed to LIB (13). It is known that the combined pathological presence of neurotoxic Aβ and tau may predict worsened long-term outcomes in blast-exposed patients, as they have been explained to exacerbate one another; for instance, with the help of tau, Aβ triggers neurodegeneration (125). Many aspects of this pathology and related mechanisms remain unclear in the context of blast exposure, and translational research is beginning to provide insight using reliable blast models and transgenic animals.

Our preclinical, open-field mouse model utilizing 46.7 kPa LIB exposure showed significantly increased tau at 7- and 30-days, and significantly increased phosphorylated tau (p-tau), Aβ40, and Aβ42 at 30 days after injury (20). Significantly increased p-tau and p-tau/tau ratio occurred in the brains of mice exposed to 82 kPa, measured at 3- and 24-h after injury (48). Elevated p-tau in the hippocampus of mice exposed to 108.9 kPa at 24 h following exposure has been reported, whereas the aberrant tau species remained elevated up to 30 days following injury (81). In a recent study, Garcia et al. subjected APP/presenilin 1 transgenic mice (APP/PS1 Tg) to repetitive low-level blast exposures (34.5 kPa) for three times per week over 8 weeks; this experimental model was designed to mimic subclinical blast exposures (126). They found, paradoxically, that if initiated at 20 weeks of age, repetitive exposures reduced anxiety and improved cognition and social interactions in APP/PS1 Tg mice, returning behavioral parameters to normal levels of non-exposed wild type mice. Repetitive LIB exposure was less effective at improving behavioral deficits in APP/PS1 Tg mice when administered at 36 weeks of age. While amyloid plaque loads were unchanged, Aβ42 levels and Aβ oligomers were reduced in the brain of mice exposed to repetitive LIB exposures initiated at 20 weeks of age. These levels did not directly correlate with behavioral parameters in individual animals. These non-intuitive results suggest unique, age-dependent genetic mechanisms that relate to blast-induced neuropathology. It is noteworthy that studies focusing on tau pathology in repetitive concussive TBI mouse models demonstrated discordant findings (127).

Overall, preclinical studies have provided valuable insights into the pathogenesis of blast induced TBI. However, more comprehensive preclinical investigations using standardized animal models are needed to better reproduce the clinical features and trajectory of human blast-TBI.

## Biomarker Development and Treatment Approaches for Blast-Induced TBI

Numerous overviews on TBI biomarkers provide essential information on the reliability of quantitative biomarkers and their diagnostic and prognostic accuracy in different TBI severities (128–131). Currently, most blood biomarkers target breakdown products generated by neuronal injury (UCH-L1, NSE, tau, and NfL), astroglial response (S100B and GFAP), neurovascular impairment (occludins, claudins, and von Willebrand factor), and inflammation (proinflammatory cytokines) (131). A biomarker test that was FDA-approved in 2018, known as The Brain Trauma Indicator, measures acute levels of UCH-L1 and GFAP in concussed individuals and provides the hope of reducing the need for unnecessary CT scans by prediction of intracranial lesions (132). A less mainstream approach to identify biomarkers involves the study of mammalian hair follicles that demonstrated unique subnetworks of miRNA following mTBI (133). As considerable inconsistencies exist pertaining to blast TBI, one of the main research goals of preclinical blast TBI studies is to aid the development of assays for highly reliable blood biomarkers. This goal might be achieved using better standardization of blast models with alignment of human and animal biology (38, 134). Biomarker research often guides the development and implementation of therapeutic strategies (135). Consequently, preclinical studies offer the potential to improve the quality and reliability of brain-related biomarkers as measures of disease severity, prognosis, and responses to treatment.

Accumulating data show a need for preclinical models to test preventive and/or treatment strategies. Several preclinical studies might serve as steppingstones for future clinical research. For example, a cannabinoid type-2 (CB2) receptor inverse agonist (SMM-189), used to treat blast-induced mTBI, significantly reduced neuronal injury, neurophysiological abnormalities, and functional deficits (136). Other studies specifically target glutamate receptors for LIB injury treatment. A study focusing on comorbid blast-induced mTBI used BCI-838 (MSGS0210), a Group II metabotropic glutamate receptor (mGluR2/3) antagonist (BCI-838), which reduced PTSD-like behavior, anxiety, fearful behavior, and long-term recognition memory impairment (104). Memantine remains among treatment options, exhibiting a potential to mitigate neuropathological and behavioral outcomes in mTBI and lessen PTSD-like behavior (137–140). As no FDA-approved treatments for TBI exist, this article provides a preview of opportunities for therapeutic research using preclinical modeling platforms.

## Future Directions

Preclinical blast research faces multiple challenges. Among these are lack of comprehensive data on austere environments with limited access to clinical data including human autopsy findings. After decades of research, it remains unclear whether the neuropathological changes caused by concussive or sub-concussive blast exposures are injury-specific or identical to impact / blast-induced TBIs. While preclinical studies show promising approaches to prevention and treatment of blast mTBI, we remain far from their clinical translation. A need exists to develop preclinical TBI common data elements (CDE) to systematically translate preclinical findings to blast injury in human. At the same time clinically relevant health impairments in Veterans and Service members should guide development of preclinical models. Using the multiple parameters incorporated into preclinical TBI CDEs with verification check-points ensuring clinical relevance will align pre-clinical research with clinical reality.

Improvement of the currently existing set of preclinical CDEs could be achieved by advanced technology such as the neurobehavioral assessment system using home-cage environment, multi-modal imaging, and high-throughput digital pathology. One of the methodological challenges in generating reliable and clinically relevant information about animals' behavior is use of conventional behavioral assessments on examining rodent activity during sleep phase. HCM approach, which captures the animal's behavior over multiple sleep- and wake-phases offers important opportunities. Non-invasive, real-time, *in vivo* molecular imaging might assume larger role in measuring brain changes after LIB exposure. Improved sensitivity of *in vivo* imaging could delineate demyelination, axonal/white matter degeneration such as DAI, neurofibrillary tangles and/or inclusion body formation, energy metabolisms, and enzymatic proteolysis. The use of transgenic animals might also offer important possibilities to mimic human susceptibilities.

## Conclusions

LIB exposure is common during military training and combat. Individuals present with varying clinical disorders and comorbidities after LIB exposure. Standardized preclinical animal models enhance insights into biological mechanisms, neurobehavioral outcomes, and cellular pathology, illustrated in Figure 1. These models, properly aligned with clinical injury scenarios, can guide future Veteran and military-centered clinical research strategies. Continuous feedback between preclinical research and clinicians is required to achieve availability of sensitive, specific biomarkers for diagnosis and prognosis of blast-induced TBI, improved treatments and rehabilitative strategies.

## Author Contributions

ZG initiated the research. HRS, SC, and ZG wrote the paper. HS, JC, IC, DC, and RD edited the paper and provided clinical translation insights. All authors contributed to the article and approved the submitted version.

## Funding

This publication was made possible in part by funding from the Department of Veterans Affairs Offices of Research & Development (VA ORD) the Biomedical Laboratory Research and Development (BLR&D) Collaborative Merit Review for TBI Research Program (I01 BX004313-01A1), and the Interagency Resource Coordinating Center (IRCC) PRECISE-TBI: PRE Clinical interagency research resource-TBI, the Education Core for deployment-related blast TBI research at Truman VA Hospital (I50 BX005878-01), as well as the DoD Congressionally Directed Medical Research Programs (CDMRP) for the Peer Reviewed Alzheimer's Research Program Convergence Science Research Award (PRARP-CSRA; W81XWH1910694, AZ180043), and the research funds of the University of Missouri (ZG).

## Author Disclaimer

Its contents are solely the responsibility of the authors and do not necessarily represent the official views of the United States government, the DoD, the United States Army, or the Department of Veterans Affairs.

## Conflict of Interest

The authors declare that the research was conducted in the absence of any commercial or financial relationships that could be construed as a potential conflict of interest.

## Publisher's Note

All claims expressed in this article are solely those of the authors and do not necessarily represent those of their affiliated organizations, or those of the publisher, the editors and the reviewers. Any product that may be evaluated in this article, or claim that may be made by its manufacturer, is not guaranteed or endorsed by the publisher.

## Abbreviations

LIB: Low-intensity blast
OFB: Open-field blast.
